# Quality of life and care burden among family caregivers of people with severe mental illness: mediating effects of self-esteem and psychological distress

**DOI:** 10.1186/s12888-022-04289-0

**Published:** 2022-10-31

**Authors:** Wan-Lin Cheng, Chih-Cheng Chang, Mark D. Griffiths, Cheng-Fang Yen, Jiun-Horng Liu, Jian-An Su, Chung-Ying Lin, Amir H. Pakpour

**Affiliations:** 1 Department of Psychiatry, Chi Mei Medical Center, Tainan, Taiwan; 2 Department of Health Psychology, Chang Jung Christian University, Tainan, Taiwan; 3 International Gaming Research Unit, Psychology Department, Nottingham Trent University, Nottingham, UK; 4 Department of Psychiatry, School of Medicine College of Medicine, Kaohsiung Medical University, Kaohsiung, Taiwan; 5 Department of Psychiatry, Kaohsiung Medical University Hospital, Kaohsiung, Taiwan; 6 College of Professional Studies, National Pingtung University of Science and Technology, Pingtung, Taiwan; 7 Department of Psychiatry, Chi Mei Medical Center, 201 Taikang Vil, Liuying Dist, 736 Liouying, Tainan City, Taiwan; 8 Department of Psychiatry, Chang Gung Medical Foundation, Chiayi Chang Gung Memorial Hospital, Chiayi, Taiwan; 9 School of Medicine, Chang Gung University, Taoyuan, Taiwan; 10 Department of Nursing, Chang Gung Institute of Technology, Taoyuan, Taiwan; 11 Institute of Allied Health Sciences, College of Medicine, National Cheng Kung University, Tainan, Taiwan; 12 Department of Occupational Therapy, College of Medicine, National Cheng Kung University, Tainan, Taiwan; 13 Department of Public Health, College of Medicine, National Cheng Kung University, Tainan, Taiwan; 14 Biostatistics Consulting Center, National Cheng Kung University Hospital, College of Medicine, National Cheng Kung University, Tainan, Taiwan; 15 Department of Nursing, School of Health and Welfare, Jönköping University, Jönköping, Sweden; 16 Institute of Allied Health Sciences, Departments of Occupational Therapy and Public Health, and Biostatistics Consulting Center, College of Medicine, National Cheng Kung University Hospital, National Cheng Kung University, 1 University Rd, 701401 Tainan, Taiwan

**Keywords:** Family caregiver, Schizophrenia, Major depressive disorder, Bipolar disorder, Quality of life, Care burden, Self-esteem

## Abstract

**Background:**

Family caregivers are important allies for healthcare providers in facilitating the recovery process among people with mental illness (PWMI). The present study examined the factors associated with quality of life (QoL) among family caregivers of PWMI.

**Methods:**

A multi-center cross-sectional survey was conducted. Family caregivers of people with schizophrenia, major depressive disorder, and bipolar disorder were recruited using convenience sampling. A survey assessing their QoL, depression, anxiety, and self-esteem was completed with self-rated psychometric scales including the Rosenberg Self-Esteem Scale, Caregiver Burden Inventory, Taiwanese Depression Questionnaire, Beck Anxiety Inventory, and World Health Organization Quality of Life Instrument Short Form. A mediation model was constructed with QoL as the dependent variable, care burden as the independent variable, and psychological distress (including depression and anxiety) with self-esteem as mediating variables.

**Results:**

Family caregivers of people with schizophrenia had worse QoL compared with counterparts of people with major depression and bipolar disorder. The sociodemographic of both caregivers and PWMI had less impact on QoL when psychological factors were considered. Caregivers with lower self-esteem, higher levels of psychological distress, and heavier care burdens had poorer QoL. Care burden had a significant total effect on QoL. Both self-esteem and psychological distress were significant mediators.

**Conclusion:**

The findings indicated that caregivers’ psychological health and care burden influenced their QoL. Interventions that target family caregivers’ self-esteem and psychological distress may attenuate the effect from care burden, and further improve their QoL.

## Introduction

Caring for people with mental illness (PWMI), particularly severe conditions such as schizophrenia, major depression, and bipolar disorder, is an ongoing and challenging process for the family caregivers [1]. In the present study, family *caregivers* were defined as the family members who take care of the PWMI without payment irrespective of whether they are primary or secondary caregivers. *Care burden* was defined as the load that caregivers subjectively perceived (e.g., obstacles involving physical, psychological, social, and financial problems) during the periods of caring for their ill relative. Increases in deinstitutionalization mean that family caregivers play a more vital role in recovery because they shoulder the burden of this process. There are complex requirements for PWMI and their family caregivers to reintegrate into the community. For example, families need to be facilitated with coping skills through professional education to avoid troublesome situations, such as disturbing, strange, or aggressive behaviors. Additionally, families and PWMI need a psychiatric rehabilitation plan with a shared understanding of the expectations. Financial or social support should be provided due to the impaired occupational and vocational functions of PWMI [2].

Quality of life (QoL) is an important concept that mental health professionals should consider among the caregivers of family members with severe mental illness. QoL is an individual’s perceived position in life related to their goals, expectations, standards, and concerns. It includes different dimensions (e.g., physical, and mental health, social relationships, and supportive environments). Moreover, this perception is considered in the context of the culture and value systems in which individuals live [3]. Poor QoL among caregivers may compromise family functioning (e.g., decision-making for therapeutic plans and emotional support). This indirectly affects the health of PWMI [2, 4]. Therefore, it is crucial to discuss the QoL of caregivers.

Prior evidence has demonstrated poorer QoL among the caregivers of PWMI than in the general population [4]. Previous studies have investigated the potential explanatory factors for this phenomenon and have reported the QoL of caregivers is predicted by some patient-dependent factors. For example, higher disease severity, including more severe psychosis or mood symptoms and more troublesome behaviors (i.e., aggressive behavior and suicidal attempts), lead to a heavier care burden and poorer QoL among caregivers [5, 6]. Additionally, male PWMI who may have earlier onset of disease and more impaired functions, tend to be associated with lower caregiver QoL [7].

Caregivers’ sociodemographic factors also play a role in their QoL. However, it is inconclusive as to whether older caregivers have a poorer QoL [4]. Female caregivers tend to have poorer QoL due to traditional family roles in different cultures [7, 8]. Parent caregivers of individuals with schizophrenia have poorer QoL compared with other family caregivers [9]. There is a higher prevalence of depression or anxiety and lower QoL scores related to lower socioeconomic status, lower education, and unemployed or financial problems among caregivers of individuals with mental and non-mental illness [8, 10–13]. Studies exploring the impact of caregiving duration on QoL have reported mix results [6, 7].

Caregivers’ own psychological and intrinsic factors, including self-esteem and psychological distress, are also strong factors associated with QoL [14, 15]. Common psychological distress among caregivers includes anxiety and depression. The associated symptoms of anxiety and depression, even if not fulfilling any of the DSM-5 diagnostic criteria, have been reported to negatively affect QoL among caregivers of PWMI and other chronic diseases (e.g., cancer and dementia) [6, 7, 15, 16].

**Fig. 1:**
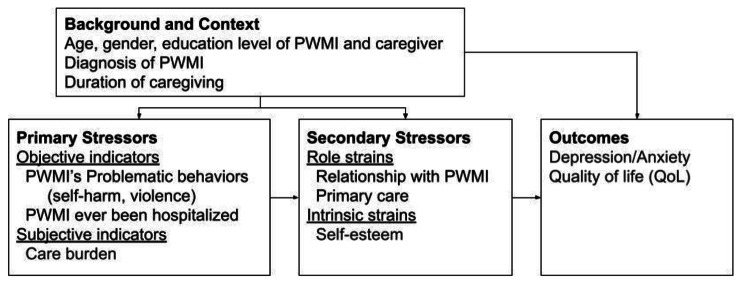
Modified version of Pearlin’s stress process model Note: This model was modified from Pearlin’s Stress Process Model [17]. In this model, all variables used in the present study were placed in their supposed position and the arrows indicate the relationships between these variables.

Pearlin [17] posited a stress process model and the present authors modified this (Fig. 1) to explain how primary stressors (e.g., care burden), secondary stressors (e.g., self-esteem) impact on the outcomes (e.g., depression, anxiety, and QoL). More specifically, background and context were constant factors impacting primary stressors, secondary stressors, and health outcomes. In addition, primary stressors may trigger secondary stressors and further develop problems related to health outcomes. The literature also provides existing empirical evidence that supports these proposed associations. In most studies, care burden tends to be an origin of lower self-esteem and higher depression or anxiety [12, 18]. Geng, Chuang et al. [13] examined the relationship between care burden and depression, and indicated a possible pathway through heavy care burden to depression, and then depression to lower QoL. Self-esteem may mediate the association from life events to depressive symptoms [19]. Additionally, self-esteem has been proposed as a mediator in the association between care burden and QoL [20]. Much evidence has shown positive associations between care burden, anxiety, and QoL [21–23]. Therefore, it was hypothesized that there would be a similar directional relationship between anxiety and depression in the present study (i.e., a heavy care burden may lead to anxiety, and then to lower QoL).

**Fig. 2:**
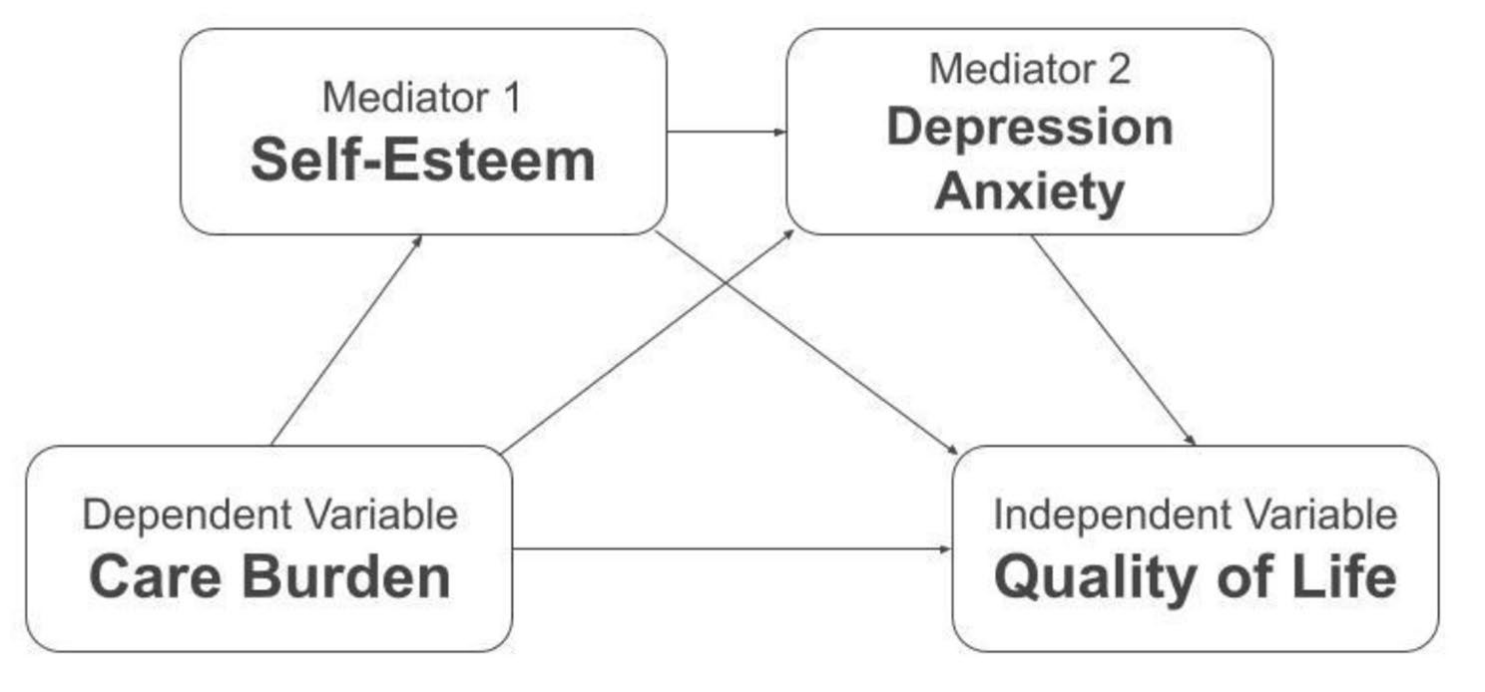
Proposed mediation model for the present study Note: Care burden, self-esteem, depression, and anxiety were proposed as having direct impacts on quality of life. Care burden was also proposed as affecting quality of life via self-esteem only, or via depression/anxiety only, or via self-esteem and depression/anxiety.

The sociodemographic factors of caregivers and PWMI are mostly unchangeable. However, care burden, self-esteem, and psychological distress are possible areas for intervention. While care burden, self-esteem, and psychological distress are predictors of caregivers’ QoL, the relationship between these factors remains unknown. Therefore, the present study examined the factors associated with QoL in the family caregivers of PWMI. Moreover, the present study proposed a mediation model (Fig. 2), which was adapted from Pearlin’s stress process model [17], using the caregivers’ care burden, self-esteem, psychological distress (i.e., depression and anxiety), and QoL. In this mediation model, care burden was considered as the primary stressor; low self-esteem was considered as a secondary stressor; and depression, anxiety, QoL were the health outcomes.

According to the mediation model adapted from the Pearlin’s stress process model, the research question of the present study concerned how care burden as a primary stressor is associated with the secondary stressor of self-esteem and health outcomes of psychological distress and QoL. The specific hypotheses based on the research question were that (i) care burden would be significantly associated with self-esteem, psychological distress, and QoL; (ii) self-esteem would be significantly associated with psychological distress and QoL; and (iii) self-esteem and psychological distress would be significant mediators in the association between care burden and QoL.

## Methods

The present study comprised a cross-sectional survey targeting families of PWMI currently receiving medical services. The participants were recruited from Chi Mei Medical Center, Chang Gung Memorial Hospital, and Kaohsiung Medical University Chung-Ho Memorial Hospital. Psychiatrists from each recruiting site invited participants who fulfilled the inclusion criteria from the outpatient department, acute or chronic ward, and home care service. The participants were then introduced to research assistants who explained the content of the survey and precautions in detail. During the study period, the research assistants provided consultation to all participants and reading support for participants with lower literacy to ensure reliability. There is no information on how many eligible participants were invited by the psychiatrists. However, for those who were transferred by the psychiatrists to participate in the present study, none of them refused to participate.

### Participants

Participants were eligible to participate if they: (i) were caregivers with at least one family member with schizophrenia, major depressive disorder, or bipolar disorder diagnosed by psychiatrists; (ii) were older than 20 years of age; (iii) were able to communicate using spoken Chinese (i.e., Mandarin or Taiwanese), or written Chinese (i.e., traditional Chinese characters); and (iv) voluntarily agreed to participate in the study.

### Measures

#### Rosenberg Self-Esteem Scale (RSES)

The 10-item RSES was used to assess self-esteem. A higher mean score indicates a higher level of self-esteem. All items (e.g., “*On the whole, I am satisfied with myself”*) are rated on a four-point Likert scale from 1 (*strongly disagree*) to 4 (*strongly agree*). Negatively worded items are reverse coded. The psychometric evidence for this scale is satisfactory. For example, it has adequate internal consistency (*α* = 0.77) and test-retest reliability (*r* = 0.63–0.85) [24–26]. Moreover, its factor structure was supported in a Taiwanese sample [27]. The internal consistency in the present study was very good (*α* = 0.85).

#### Caregiver Burden Inventory (CBI)

The 24-item CBI was used to assess care burden. A higher mean score indicates a higher level of care burden. All items (e.g., *“I do not have enough sleep”*) are rated using a five-point Likert scale from 0 (*strongly disagree*) to 4 (*strongly agree*) [28]. The psychometric evidence of the Taiwanese CBI is satisfactory. For example, it has very good internal consistency (*α* = 0.91) and concurrent validity [29]. The internal consistency in the present study was excellent (*α* = 0.93).

#### Taiwanese Depression Questionnaire (TDQ)

The 18-item TDQ was used to assess depression [30]. A higher mean score indicates a higher level of depression. The instrument was developed based on the question: “How often did you feel physical and emotional aspects during the past week?” All items (e.g., *“I feel depressed”*) are rated using a four-point Likert scale from 0 (*none or extremely few, < 1 day per week*) to 3 (*often or always, 5–7 days per week*) [30]. The psychometric evidence is satisfactory. It has adequate internal consistency (*α* = 0.90), satisfactory sensitivity (0.89), and specificity (0.92) [31]. The internal consistency in the present study was excellent (*α* = 0.93).

#### Beck Anxiety Inventory (BAI)

The BAI was used to assess anxiety. A higher mean score indicates a higher level of anxiety. All items (e.g., *“numbness or tingling”*) are rated using a four-point Likert scale from 0 (*not at all*) to 3 (*severely: I could barely stand it*). It has good internal consistency (*α* = 0.92), test-retest reliability (*r* = 0.75), and known-group validity [32]. The BAI has shown acceptable internal consistency and confirmed known-group validity in a Taiwanese sample [33]. The internal consistency in the present study was excellent (*α* = 0.91).

#### World Health Organization Quality of Life Instrument Short Form (WHOQOL-BREF) Taiwan Version

The 28-item WHOQOL-BREF Taiwan Version was used to assess QoL. It was shortened from the WHOQOL long form for Taiwanese. In addition to the original 26 items from the standard WHOQOL-BREF, two items were added according to national surveys to reflect Taiwanese culture. Therefore, the WHOQOL-BREF Taiwan version contains 28 items classified into the same four domains as the standard WHOQOL-BREF: physical health (PHY: seven items), psychological health (PSY: six items), social relationships (SR: four items), environment (ENV: nine items), and general health (two items). Each item (e.g., *“How would you rate your quality of life?”*) is rated on a five-point Likert scale from 1 (*not at all*) to 5 (*completely*) and there are four types of scale descriptors (capacity, frequency, intensity, and evaluation). The domain scores are calculated using a linear transformation from the original five-point Likert scale. The domain scores are between 4 and 20 with a higher score indicating a better of QoL on that domain [34]. The WHOQOL-BREF Taiwan version has shown acceptable internal consistency (*α* = 0.70–0.77 for four domains) and validity (0.51–0.64 for inter-domain correlations) [34]. The internal consistency in the present study was excellent (*α* = 0.93).

### Statistical analysis

After using descriptive analyses to summarize the participants’ characteristics and their outcome measure performance, WHOQOL-BREF scores between groups (i.e., caregivers caring for a family member with schizophrenia, bipolar disorder, and major depressive disorder) were compared using analyses of variance (ANOVAs) with Bonferroni correction. Next, several hierarchical regression analyses were constructed to examine the potential factors associated with QoL. In the hierarchical regression models, each domain mean score of the WHOQOL-BREF was the dependent variable (i.e., a total of four sets of hierarchical regression models on physical health, psychological health, social relationships, and environment QoL). The demographic and clinical characteristics of PWMI were independent variables in Model 1; the demographic characteristics of caregivers were added as independent variables in Model 2; and the psychological factors of caregivers (i.e., self-esteem, care burden, depression, and anxiety) were added as independent variables in Model 3.

To examine the sequentially mediated effects of self-esteem and depression (and anxiety) in the association between care burden and QoL, Hayes’ Process Macro Model 6 [35] was used. In the mediation model (Fig. 2), care burden was the independent variable. Each domain of QoL were dependent variables. Self-esteem and psychological distress (depression and anxiety) were the first and the second mediators, respectively. Moreover, the demographic characteristics of PWMI and their caregivers were controlled for in the mediation model because prior evidence has shown that they are important confounders in caregiver QoL [4, 7, 11]. The mediated effects were tested using the bootstrapping method to determine whether they were significant. More specifically, 5,000 bootstrapping samples with bias corrected confidence intervals were generated. If the two limits of 95% confidence interval do not cover 0, the mediated effect is supported [35]. There were almost no missing data in the present study (< 0.1%) and missing values were accounted for in the data analysis by using the pairwise deletion method. All statistical analyses were conducted using SPSS version 26.

## Results

### Descriptive analysis

In total, 459 dyads of caregivers and PWMI were recruited. Most participants were middle aged (mean age: 50.5 years [SD ± 17.1] for PWMI and 53.3 years [SD ± 13.5] for caregivers). Three-quarters of the caregivers were married (76%). However, less than half of PWMI were married (48.4%). Both caregivers and PWMI had a mean education of approximately nine years. The mean duration of caregiving was 10.3 years (SD ± 9.0). Among the PWMI, the diagnoses were schizophrenia (46.8%), bipolar disorder (18.5%), and depressive disorder (34.6%). The mean age of first treatment was 38.6 years. Half of the PWMIs had ever been admitted to acute or chronic psychiatric wards (50.5%) and 22% had undergone compulsory admission to a psychiatric acute ward. A quarter of PWMI had reported a previous suicide attempt or self-harm behavior (26%). The mean scale scores for caregivers, including self-esteem, care burden, depression, anxiety, and WHOQOL, are shown in Table 1.

**Table 1:** Participant characteristics

			N or MEAN	% or SD
**People with mental illness (PWMI)**				
	Age (in years)		50.5	17.1
	Gender			
		Male/Female	208/251	45.3/54.7
	Marital status			
		Married, cohabiting, remarried	222	48.4
		Widowed, separated, divorced	82	17.9
		Single	155	33.8
	Education (in years)		9.1	5.0
	Diagnosis			
		Schizophrenia	215	46.8
		Bipolar disorder	85	18.5
		Major depressive disorder	159	34.6
	Age of first treatment (year)		38.6	18.6
	Psychiatric hospitalization			
		Yes/No/Unknown	232/225/2	50.5/49/0.5
	Compulsory admission			
		Yes/No/Unknown	101/350/8	22/76.3/1.7
	Suicidal attempt/behavior			
		Yes/No/Unknown	121/333/5	26.4/72.5/1.1
**Caregiver’s demographic characteristics**				
	Age (in years)		53.3	13.5
	Gender			
		Male/Female	224/235	48.8/51.2
	Marital status			
		Married, cohabiting, remarried	349	76
		Widowed, separated, divorced	59	12.9
		Single	50	10.9
		Others	1	0.2
	Education (in years)		9.9	4.7
	Relationship with PWMI			
		Parent/Spouse/Others	136/148/175	29.6/32.2/38.2
	Duration of caregiving (in years)		10.3	9.0
	Primary caregiver			
		Yes/No	393/66	85.6/14.4
**Caregiver’s psychological characteristics**				
	Self-esteem		29.1	4.9
	Care burden		33.1	19.3
	Depression		10.1	9.5
	Anxiety		26.1	6.9
**Quality of life (WHOQOL-BREF Taiwan Version)**				
	Physical health		14.1	2.6
	Psychological		12.6	2.8
	Social relationships		13.4	2.6
	Environment		13.4	2.6

### Main findings in hierarchical regression analysis

The results of the hierarchical regression models are shown in Table 2. Families with lower self-esteem, higher depression, and heavier care burden were associated with poorer QoL in the Model 3 of the hierarchical regression analysis. In addition, Model 1 of the hierarchical regression analysis showed that the following variables were significant to at least one of the low levels of QoL domains: caring for a family member with bipolar disorder (compared to schizophrenia), male gender, younger age, fewer years of education, and psychiatric hospitalization ever. However, these differences were diminished when the caregiver demographics were included in Model 2, except for psychiatric hospitalization ever (*β* = −0.13, *p <* 0.05) in the environmental domain. The following variables of caregiver demographics were related to at least one of the low levels of QoL domains: being of younger age, having fewer years of education, and being a parent caregiver.

**Table 2:** Predictors of the WHOQOL utilizing a hierarchical regression model

		Domain1 Physical health						Domain 2 Psychological health						Domain 3 Social relationships						Domain4 Environment					
		Model 1		Model 2		Model 3		Model 1		Model 2		Model 3		Model 1		Model 2		Model 3		Model 1		Model 2		Model 3	
		β	*t*	β	*t*	β	*t*	β	*t*	β	*t*	β	*t*	β	*t*	β	*t*	β	*t*	β	*t*	β	*t*	β	*t*
**PWMI’s demographic and illness characteristics**																									
	Age	0.18	1.85	0.10	0.66	0.06	0.53	0.26**	2.66	0.08	0.51	0.02	0.17	0.21*	2.08	0.03	0.21	0.00	0.00	0.35***	3.58	0.14	0.98	0.10	0.77
	Male (reference: female)	-0.12*	-2.34	-0.06	-1.12	-0.02	-0.46	-0.07	-1.39	0.00	0.03	0.04	1.08	-0.05	-0.94	-0.02	-0.29	0.01	0.13	-0.07	-1.40	-0.01	-0.27	0.01	0.11
	Married, cohabiting, remarried (reference: single)	-0.03	-0.35	-0.02	-0.22	-0.03	-0.49	-0.06	-0.84	-0.02	-0.18	-0.02	-0.37	-0.05	-0.73	0.03	0.29	0.02	0.26	0.02	0.25	0.10	1.05	0.09	1.13
	Widowed, separated, divorced (reference: single)	-0.05	-0.73	-0.08	-1.12	-0.03	-0.66	-0.05	-0.77	-0.05	-0.63	0.00	0.00	-0.09	-1.26	-0.05	-0.72	-0.03	-0.49	-0.01	-0.08	0.07	0.95	0.09	1.51
	Number of years of education	0.16*	2.31	0.02	0.29	-0.01	-0.22	0.20**	2.89	0.08	1.08	0.05	0.89	0.04	0.64	-0.07	-0.97	-0.09	-1.44	0.26***	3.93	0.10	1.32	0.07	1.21
	Bipolar disorder (reference: schizophrenia)	0.11*	2.09	0.09	1.64	0.05	1.21	0.09	1.70	0.08	1.45	0.02	0.64	0.05	0.93	0.04	0.72	-0.01	-0.20	0.06	1.17	0.05	0.91	0.01	0.30
	Major depressive disorder (reference: schizophrenia)	0.00	0.02	-0.04	-0.55	-0.05	-0.90	0.08	1.10	0.05	0.72	0.04	0.71	-0.09	-1.23	-0.10	-1.42	-0.13*	-1.99	0.05	0.64	0.01	0.14	-0.02	-0.39
	Age of treatment initiation	0.05	0.54	0.02	0.14	0.04	0.41	0.00	0.04	-0.01	-0.07	0.02	0.21	-0.02	-0.20	-0.09	-0.66	-0.08	-0.61	0.00	0.03	-0.03	-0.21	0.01	0.07
	Psychiatric hospitalization ever (reference: never)	-0.14*	-2.21	-0.09	-1.43	-0.06	-1.42	-0.10	-1.61	-0.07	-1.08	-0.02	-0.45	-0.15*	-2.27	-0.11	-1.75	-0.06	-1.07	-0.15*	-2.45	-0.13*	-2.14	-0.08	-1.54
	Suicidal attempt/behavior ever (reference: never)	-0.01	-0.23	-0.02	-0.49	0.05	1.47	-0.06	-1.19	-0.07	-1.51	-0.01	-0.22	-0.02	-0.33	-0.03	-0.57	0.00	0.08	0.01	0.17	0.00	0.03	0.04	1.04
	Compulsory admission ever (reference: never)	0.05	0.85	0.00	0.05	0.05	1.31	0.00	0.03	-0.04	-0.64	0.00	0.10	-0.04	-0.62	-0.07	-1.32	-0.05	-1.02	0.09	1.69	0.06	1.10	0.08	1.76
		F = 2.96**, R2 = 0.07a						F = 3.12***, R2 = 0.08a						F = 1.88*, R2 = 0.05a						F = 4.81***, R2 = 0.11a					
**Caregiver’s demographic characteristics**																									
	Age			0.02	0.22	-0.08	-1.41			0.15	1.89	0.04	0.83			0.22**	2.77	0.14*	2.11			0.26**	3.49	0.19**	2.91
	Male (reference: female)			0.03	0.61	-0.02	-0.49			0.05	1.05	0.00	0.03			-0.06	-1.17	-0.10*	-2.17			-0.02	-0.37	-0.05	-1.24
	Married, cohabiting, remarried (reference: single)			0.03	0.39	0.05	0.96			-0.03	-0.34	0.00	0.00			0.03	0.37	0.04	0.62			0.01	0.07	0.03	0.48
	Widowed, separated, divorced (reference: single)			-0.13	-1.78	-0.02	-0.35			-0.09	-1.25	0.01	0.28			-0.02	-0.22	0.05	0.68			-0.11	-1.49	-0.05	-0.72
	Number of years of education			0.25***	3.92	0.09*	2.02			0.24***	3.86	0.07	1.51			0.23***	3.55	0.09	1.53			0.31***	5.07	0.19***	3.56
	Parent (reference: others)			-0.07	-0.76	-0.02	-0.33			-0.18*	-2.00	-0.14*	-2.23			-0.21*	-2.35	-0.18*	-2.28			-0.08	-0.87	-0.04	-0.59
	Spouse (reference: others)			-0.11	-1.34	-0.02	-0.30			-0.15	-1.76	-0.05	-0.92			-0.17	-1.97	-0.10	-1.32			-0.08	-1.02	-0.03	-0.43
	Duration of caregiving (in years)			-0.01	-0.13	-0.02	-0.33			0.00	-0.01	0.00	-0.05			-0.03	-0.36	-0.03	-0.45			0.00	-0.01	0.01	0.22
	Primary caregiver (reference: no)			-0.02	-0.37	0.01	0.25			-0.02	-0.33	0.02	0.41			-0.07	-1.26	-0.04	-0.87			-0.06	-1.16	-0.03	-0.61
		F = 4.18***, R^2^ = 0.17b, ΔR2 = 0.10						F = 3.70***, R^2^ = 0.16b, ΔR^2^ = 0.08						F = 2.59***, R^2^ = 0.11b, ΔR^2^ = 0.07						F = 4.88***, R^2^ = 0.20b, ΔR^2^ = 0.08					
**Caregiver’s self-esteem, burden and distress**																									
	Self-esteem					0.20***	4.66					0.28***	6.62					0.29***	5.26					0.17**	3.34
	Care burden					-0.09*	-2.05					-0.13**	-2.99					-0.12*	-2.22					-0.25***	-4.81
	Depression					-0.43***	-7.86					-0.44***	-8.26					-0.22**	-3.23					-0.24***	-3.68
	Anxiety					-0.09	-1.85					0.00	-0.06					0.01	0.14					0.09	1.52
		F = 23.24***, R^2^ = 0.58c, ΔR2 = 0.41						F = 25.55***, R^2^ = 0.61c, ΔR^2^ = 0.45						F = 8.82***, R^2^ = 0.35c, ΔR^2^ = 0.23						F = 11.73***, R^2^ = 0.41c, ΔR^2^ = 0.22					
		a Adjusted R2 = 0.05. b Adjusted R2 = 0.13. c Adjusted R2 = 0.56. *p < 0.05, **p < 0.01, ***p < 0.001.						a Adjusted R2 = 0.05. b Adjusted R2 = 0.11. c Adjusted R2 = 0.58. *p < 0.05, **p < 0.01, ***p < 0.001.						a Adjusted R2 = 0.02. b Adjusted R2 = 0.07. c Adjusted R2 = 0.31. *p < 0.05, **p < 0.01, ***p < 0.001.						a Adjusted R2 = 0.09. b Adjusted R2 = 0.16. c Adjusted R2 = 0.38. *p < 0.05, **p < 0.01, ***p < 0.001.					

### Main findings in sequential mediation model

**Fig. 3:**
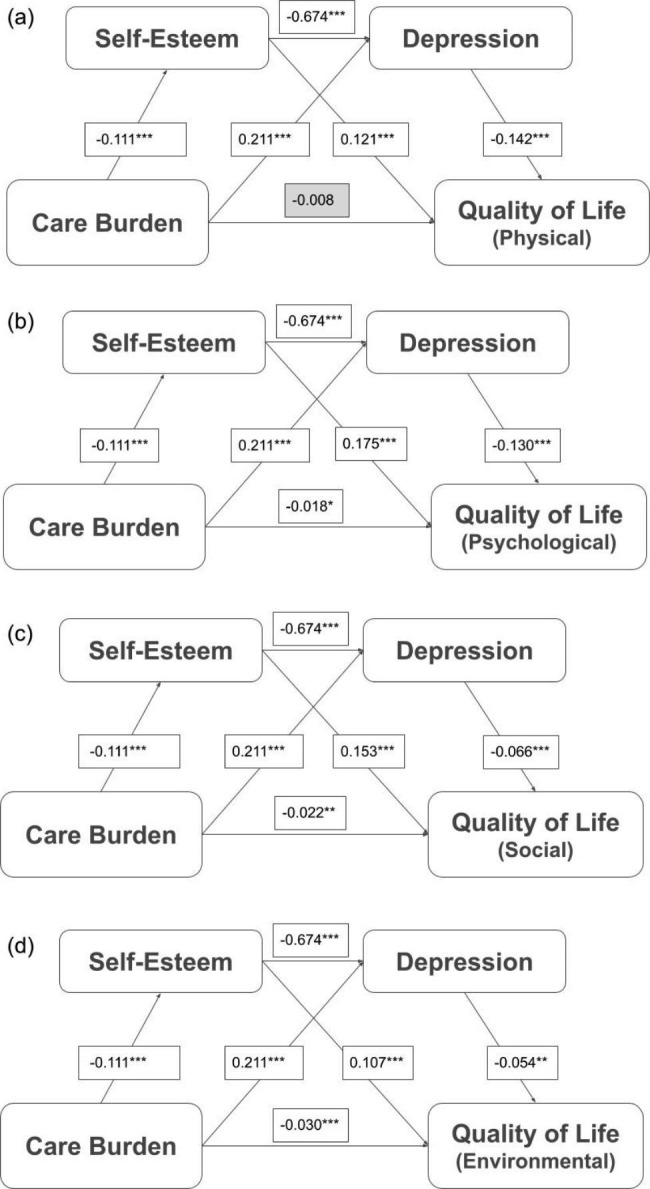
Results of sequential mediation model describing the mediator roles of self-esteem and depression in the association between care burden and quality of life Note: Mediated effects of the level of quality of life (comprising four domains: physical health, psychological health, social relationships, and environment) on the associations between care burden, self-esteem, and depression. All the models used 5,000 bootstrapping resamples and controlled for people with mental illness (PWMI) diagnosis, psychiatric hospitalization, age of PWMI and caregiver, gender of PWMI and caregiver, and education years of PWMI and caregiver. All coefficients and effects are unstandardized coefficients/effects. All path coefficients were significant Abbreviations: PHY = physical health; PSY = psychological health; SR = social relationships; ENV = environment. *p < 0.05, **p < 0.01, ***p < 0.001

**Fig. 4:**
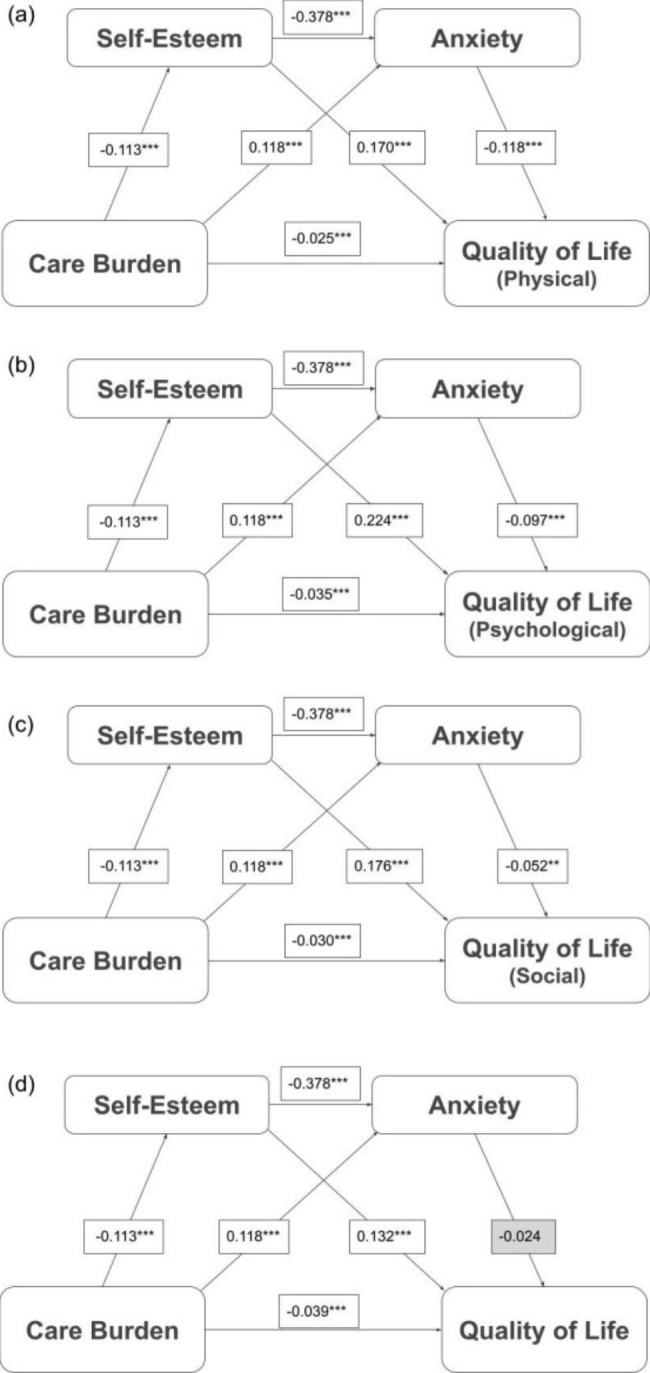
Results of sequential mediation model describing the mediator roles of self-esteem and depression in the association between care burden and quality of life Note: Mediated effects of the level of quality of life (comprising four domains: physical health, psychological health, social relationships, environment) on the associations between care burden, self-esteem, and anxiety. All the models used 5,000 bootstrapping resamples and controlled for diagnosis of people with mental illness (PWMI), psychiatric hospitalization, age of PWMI and caregiver, gender of PWMI and caregiver, and education years of PWMI and caregiver. All coefficients and effects are unstandardized coefficients/effects. All path coefficients were significant Abbreviations: PHY = physical health; PSY = psychological health; SR = social relationships; ENV = environment. *p < 0.05, **p < 0.01, ***p < 0.001

The results of the sequential mediation model are presented in Fig. 3 (mediators of self-esteem and depression) and Fig. 4 (mediators of self-esteem and anxiety). The mediation model showed that care burden had a significant total effect on QoL in all four domains (Tables 3 and 4). The pathway analysis of care burden, self-esteem, depression, and QoL showed that almost all direct associations (care burden to self-esteem, depression and QoL; self-esteem to depression, QoL; depression to QoL) were significant, except on PHY (Fig. 3). Significant indirect effects were observed in the association between care burden and QoL via self-esteem only (coefficient = − 0.014; 95% CI = − 0.020, − 0.008 [PHY]; coefficient = − 0.020; 95% CI = − 0.027, − 0.013 [PSY]; coefficient = − 0.017; 95% CI = − 0.025, − 0.010 [SR]; coefficient = − 0.012; 95% CI = − 0.019, − 0.006 [ENV]), depression only (coefficient = − 0.030; 95% CI = -0.039, − 0.022 [PHY]; coefficient = − 0.028; 95% CI =-0.036, − 0.020 [PSY]; coefficient = − 0.014; 95% CI = − 0.021, -0.008 [SR]; coefficient = − 0.011; 95% CI = -0.018, − 0.005 [ENV]), or self-esteem and depression sequentially (coefficient = − 0.011; 95% CI = − 0.015, − 0.007 [PHY]; coefficient = − 0.010; 95% CI = − 0.014, − 0.006 [PSY]; coefficient = − 0.005; 95% CI = − 0.008, − 0.003 [SR]; coefficient = − 0.004; 95% CI = − 0.007, − 0.002 [ENV]). These results indicate that self-esteem and depression were mediators of the association between care burden and QoL (Table 3).

**Table 3:** Results of sequential mediation model showing total, direct, and indirect effects on quality of life (depression)

Variable	Effect	Standard error	*t*	*p*-value
**Physical health**				
Total effect of care burden	-0.062	0.006	-10.92	< 0.001
Direct effect of care burden	-0.008	0.006	-1.39	0.166
Indirect effect of care burden	Effect	Bootstrapping SE	Bootstrapping LLCI	Bootstrapping ULCI
Total indirect effect of care burden	-0.054	0.005	-0.064	-0.045
Indirect effect via self esteem	-0.014	0.003	-0.020	-0.008
Indirect effect via depression	-0.030	0.004	-0.039	-0.022
Indirect effect via self-esteem and depression	-0.011	0.002	-0.015	-0.007
**Psychological health**				
Total effect of care burden	-0.075	0.006	-12.86	< 0.001
Direct effect of care burden	-0.018	0.006	-3.16	0.002
Indirect effect of care burden	Effect	Bootstrapping SE	Bootstrapping LLCI	Bootstrapping ULCI
Total indirect effect of care burden	-0.057	0.005	-0.067	-0.047
Indirect effect via self esteem	-0.020	0.003	-0.027	-0.013
Indirect effect via depression	-0.028	0.004	-0.036	-0.020
Indirect effect via self-esteem and depression	-0.010	0.002	-0.014	-0.006
**Social relationships**				
Total effect of care burden	-0.058	0.005	-10.63	< 0.001
Direct effect of care burden	-0.022	0.006	-3.59	< 0.001
Indirect effect of care burden	Effect	Bootstrapping SE	Bootstrapping LLCI	Bootstrapping ULCI
Total indirect effect of care burden	-0.036	0.005	-0.046	-0.027
Indirect effect via self esteem	-0.017	0.004	-0.025	-0.010
Indirect effect via depression	-0.014	0.003	-0.021	-0.008
Indirect effect via self-esteem and depression	-0.005	0.001	-0.008	-0.003
**Environment**				
Total effect of care burden	-0.057	0.006	-10.41	< 0.001
Direct effect of care burden	-0.030	0.007	-4.58	< 0.001
Indirect effect of care burden	Effect	Bootstrapping SE	Bootstrapping LLCI	Bootstrapping ULCI
Total indirect effect of care burden	-0.027	0.005	-0.036	-0.019
Indirect effect via self esteem	-0.012	0.003	-0.019	-0.006
Indirect effect via depression	-0.011	0.003	-0.018	-0.005
Indirect effect via self-esteem and depression	-0.004	0.001	-0.007	-0.002

Abbreviations: SE = standard error; LLCI = lower limit of confidence interval; ULCI: upper limit of confidence interval

**Table 4:** Results of sequential mediation model showing total, direct, and indirect effects on quality of life (anxiety)

Variable	Effect	Standard error	*t*	*p*-value
**Physical health**				
Total effect of care burden	-0.063	0.006	-11.13	< 0.001
Direct effect of care burden	-0.025	0.006	-4.35	< 0.001
Indirect effect of care burden	Effect	Bootstrapping SE	Bootstrapping LLCI	Bootstrapping ULCI
Total indirect effect of care burden	-0.038	0.004	-0.047	-0.030
Indirect effect via self esteem	-0.019	0.004	-0.026	-0.013
Indirect effect via anxiety	-0.014	0.003	-0.019	-0.009
Indirect effect via self-esteem and anxiety	-0.005	0.001	-0.008	-0.003
**Psychological health**				
Total effect of care burden	-0.076	0.006	-13.01	< 0.001
Direct effect of care burden	-0.035	0.006	-6.00	< 0.001
Indirect effect of care burden	Effect	Bootstrapping SE	Bootstrapping LLCI	Bootstrapping ULCI
Total indirect effect of care burden	-0.041	0.005	-0.050	-0.032
Indirect effect via self esteem	-0.025	0.004	-0.033	-0.019
Indirect effect via anxiety	-0.012	0.003	-0.017	-0.007
Indirect effect via self-esteem and anxiety	-0.004	0.001	-0.007	-0.002
**Social relationships**				
Total effect of care burden	-0.058	0.006	-10.66	< 0.001
Direct effect of care burden	-0.030	0.006	-5.09	< 0.001
Indirect effect of care burden	Effect	Bootstrapping SE	Bootstrapping LLCI	Bootstrapping ULCI
Total indirect effect of care burden	-0.028	0.004	-0.037	-0.020
Indirect effect via self esteem	-0.020	0.004	-0.028	-0.013
Indirect effect via anxiety	-0.006	0.002	-0.010	-0.002
Indirect effect via self-esteem and anxiety	-0.002	0.001	-0.004	-0.001
**Environment**				
Total effect of care burden	-0.058	0.006	-10.49	< 0.001
Direct effect of care burden	-0.039	0.006	-6.22	< 0.001
Indirect effect of care burden	Effect	Bootstrapping SE	Bootstrapping LLCI	Bootstrapping ULCI
Total indirect effect of care burden	-0.019	0.004	-0.026	-0.012
Indirect effect via self esteem	-0.015	0.004	-0.022	-0.009
Indirect effect via anxiety	-0.003	0.002	-0.007	0.001
Indirect effect via self-esteem and anxiety	-0.001	0.001	-0.003	0.000

Abbreviations: SE = standard error; LLCI = lower limit of confidence interval; ULCI: upper limit of confidence interval

In the pathway analysis of care burden, self-esteem, anxiety, and QoL, the direct association between anxiety and the QoL environmental domain was not significant, while other direct associations (care burden to self-esteem, anxiety and QoL; self-esteem to anxiety, QoL; anxiety to QoL) were significant in all four QoL domains (Fig. 4). Significant indirect effects were observed in the association between care burden and QoL via self-esteem only (coefficient = − 0.019; 95% CI = − 0.026, − 0.013 [PHY]; coefficient = − 0.025; 95% CI = − 0.033, − 0.019 [PSY]; coefficient = − 0.020; 95% CI = − 0.028, − 0.013 [SR]; coefficient = − 0.015; 95% CI = − 0.022, − 0.009 [ENV]), anxiety only (coefficient = − 0.014; 95% CI = − 0.019, − 0.009 [PHY]; coefficient = − 0.012; 95% CI = − 0.017, − 0.007 [PSY]; coefficient = − 0.006; 95% CI = − 0.010, − 0.002 [SR]; coefficient = − 0.003; 95% CI = − 0.007, 0.001 [ENV]), or self-esteem and anxiety sequentially (coefficient = − 0.005; 95% CI = − 0.008, − 0.003[PHY]; coefficient = − 0.004; 95% CI = − 0.007, − 0.002 [PSY]; coefficient = − 0.002; 95% CI = − 0.004, − 0.001 [SR]; coefficient = − 0.001; 95% CI = − 0.003, 0.000 [ENV]). These results indicated that self-esteem and anxiety were mediators of the association between care burden and QoL domains, including PHY, PSY, and SR, but did not mediate care burden and ENV (Table 4).

### Findings in analyses of variance

The QoL scores between groups are shown in Table 5. The caregivers of a family member with schizophrenia had significantly lower QoL than those of a family member with bipolar or major depressive disorder in PHY (schizophrenia < bipolar disorder, *p* = 0.009; schizophrenia < major depression, *p* = 0.043), PSY (schizophrenia < major depression, *p* = 0.023), and ENV (schizophrenia < major depression, *p* = 0.002). No significant differences were found in SR between groups (*F* = 1.18, *p* > 0.05).

**Table 5:** Comparison of quality of life scores between three mental health illnesses using analysis of variance

	1. Schizophrenia			2. Bipolar disorder			3. Major depressive disorder				
	Mean	SD		Mean	SD		Mean	SD		F	Comparison
WHOQOL domain											
Physical health	13.71	2.61		14.71	2.66		14.39	2.62		5.57**	2 and 3 > 1
Psychological health	12.16	2.69		12.84	2.91		12.94	2.86		4.13*	3 > 1
Social relationships	13.27	2.64		13.77	2.46		13.51	2.66		1.18	-
Environment	12.99	2.46		13.67	2.53		13.88	2.61		6.23**	3 > 1

*p < 0.05, **p < 0.01; comparisons were made using Bonferroni adjustment

## Discussion

Currently, there are only a few studies that have conducted pathway analysis to explain the effect of care burden on QoL among caregivers of PWMI. The result of the present study supported the adapted stress process model, in which care burden affected QoL in all domains under the mediating effect of self-esteem and psychological distress. Similarly, previous studies had found that heavy care burden contributes negatively to a caregiver’s psychological health and social life [7, 22, 23]. Moreover, self-esteem is an internal protective resource that can influence subjective perceptions to events [19, 36]. Given that QoL is defined as an individual’s subjective perceptions to life, self-esteem may have a positive relationship with QoL in all domains, as shown in the present study.

This low self-esteem is also highly correlated with depressive symptoms and suicidal ideation [37]. Depression and anxiety are frequently discussed together in research due to their high comorbidity rates [22, 23, 38]. In the present study’s proposed model, depression was directly related to lower QoL in all domains and mediated care burden to QoL. Anxiety directly related to lower QoL and mediated care burden to QoL in PHY, PSY, and SR but not in ENV. Other studies have reported similar results [38, 39]. Care burden makes caregivers more vulnerable to depression and anxiety due to several factors, such as social isolation and stigmatization [22]. By contrast, negative and worrisome thinking patterns may lead caregivers to focus more on the negative aspects in life. Consequently, care burden may contribute to low QoL via depression and anxiety.

The present study showed that caregivers of a family member with schizophrenia generally had the poorest QoL when compared with those caring for family members with bipolar or major depressive disorders. Findings from the present study concurred with the accumulated evidence regarding the sociodemographic characters analyzed in the hierarchical regression model [4, 7]. Higher education and older caregivers had better QoL in the PHY, SR, and ENV domains. These caregivers tended to cope with stress better and search for social and financial resources. Parent caregivers had poorer QoL in PSY and SR most likely because they suffer more from excessive loss and guilt [9]. Previous studies have reported mixed results regarding the impact of gender on caregiver QoL [4, 7]. In the present study, male caregivers had lower QoL scores than female caregivers in SR. Further research into gender and caregivers in Taiwan is therefore needed.

Mental health among caregivers is an important issue. The present study comprised a larger sample size than previous studies and was well-structured. Consequently, the present study provides more persuasive evidence for current strategies that target the mental health of caregivers who look after PWMI. According to Pearlin’s model and the modified model presented here, management of primary stressors (i.e., care burden) and secondary stressors (i.e., low self-esteem) could improve outcomes (i.e., psychological distress and QoL). Interventions such as group supportive therapies, home-based training programs, and psychoeducation have been shown to be of benefit [40–42]. Therefore, clinicians should be aware of the impact effect of care burden on caregivers’ own life and should help them by implementing evidence-based interventions.

There are some limitations in the present study. First, mediation and hierarchical regression models were used to understand the factors associated with caregiver QoL. However, the present study provided no evidence of causal relationships because it utilized a cross-sectional study design that does not consider the effect of time. Therefore, it cannot provide evidence for causal relationships. Second, all the variables used in the present study were assessed using self-reports. Therefore, the findings are likely to be biased because of factors such as social desirability and common method variance. Future studies using different methods to collect data should be conducted to corroborate the present study’s findings. Third, the present study did not collect any information on comorbidities in the PWMI. Moreover, comorbidity was not an exclusion criterion. Therefore, it is unclear whether comorbidities could have confounded the findings. Fourth, the cohort was recruited from medical centers in southern Taiwan. Therefore, the present findings cannot be generalized to all caregivers of PWMI in Taiwan or to caregivers from other countries and cultures.

## Conclusion

The results of the present study confirm that, for caregivers, psychological factors had a strong impact on their QoL. Additionally, care burden influenced QoL in all domains, and this association was mediated by self-esteem and psychological health (depression or anxiety). Further studies are needed to confirm how these factors impact QoL. Clinicians can use self-esteem, depression, and anxiety as entry points to intervene and improve the QoL among caregivers. Interventions that target family caregivers’ self-esteem and psychological distress may attenuate the effect from care burden, and further improve their QoL.

## Data Availability

The datasets generated and/or analyzed during the current study are not publicly available due to ethic issues involving participant’s data and privacy but are available from the corresponding author on reasonable request.

## Abbreviations

PWMI: People with mental illness
QoL: Quality of life
RSES: Rosenberg Self-Esteem Scale
CBI: Caregiver Burden Inventory
TDQ: Taiwanese Depression Questionnaire
BAI: Beck Anxiety Inventory
WHOQOL-BREF: World Health Organization Quality of Life Instrument Short Form
ANOVA: Analysis of variance
PHY: Physical quality of life
PSY: Psychological quality of life
SR: Social relationships quality of life
ENV: Environmental quality of life
